# Acoustic Waveguide Eigenmode Solver Based on a Staggered-Grid Finite-Difference Method

**DOI:** 10.1038/s41598-017-17511-x

**Published:** 2017-12-13

**Authors:** Nathan Dostart, Yangyang Liu, Miloš A. Popović

**Affiliations:** 1 University of Colorado Boulder, Department of Electrical, Computer, and Energy Engineering, Boulder, 80309 USA; 2 Boston University, Department of Electrical and Computer Engineering, Boston, 02215 USA

## Abstract

A numerical method of solving for the elastic wave eigenmodes in acoustic waveguides of arbitrary cross-section is presented. Operating under the assumptions of linear, isotropic materials, it utilizes a finite-difference method on a staggered grid to solve for the acoustic eigenmodes (field and frequency) of the vector-field elastic wave equation with a given propagation constant. Free, fixed, symmetry, and anti-symmetry boundary conditions are implemented, enabling efficient simulation of acoustic structures with geometrical symmetries and terminations. Perfectly matched layers are also implemented, allowing for the simulation of radiative (leaky) modes. The method is analogous to that in eigenmode solvers ubiquitously employed in electromagnetics to find waveguide modes, and enables design of acoustic waveguides as well as seamless integration with electromagnetic solvers for optomechanical device design. The accuracy of the solver is demonstrated by calculating eigenfrequencies and mode shapes for common acoustic modes across four orders of magnitude in frequency in several simple geometries and comparing the results to analytical solutions where available or to numerical solvers based on more computationally expensive methods. The solver is utilized to demonstrate a novel type of leaky-guided acoustic wave that couples simultaneously to two independent radiation channels (directions) with different polarizations – a ‘bi-leaky’ mode.

## Introduction

Recent advances in several fields have attracted growing interest in the design of chip-scale acoustic devices that can interface with electrical and optical integrated components. Optomechanics is a prime example, where interacting acoustic and optical fields enable novel functionalities, such as ultra-sensitive quantum measurements^1,2^, narrow-linewidth lasers^3,4^, optomechanical memory^5,6^, non-reciprocity and optical diodes^7–9^, optical cooling^10–12^, phononic topological insulators^13,14^, optical amplifiers^15^, improved gravitational wave detection^16,17^, microwave filters^18^, and quantum state transfer^19–21^. The field of RF micro-electromechanical systems is another, older example where electroacoustic transduction of bulk and surface acoustic waves in acoustic resonators enables some devices which outperform conventional RF electronics. These devices include reconfigurable filters^22^, narrowband signal filtering^23^, and high quality factor (Q) resonators^24^. These devices are also being integrated into microelectronic systems for improved performance^25^. For all these acoustic wave based devices, good performance requires confining most of the acoustic energy to a small cross-sectional area (waveguides) or volume (resonators), phase-matching the acoustic wave to transducer arrays and/or optical waves, and optimizing transduction efficiency. Numerical tools for designing and simulating acoustic waveguide modes are thus necessary to enable efficient device designs, intricate nano-scale coupling schemes, and novel device architectures.

Previous work has predominantly focused on the full-wave simulation of source-driven responses in 2D and 3D domains. 3D solvers of this kind have been developed for anisotropic, heterogeneous domains using both finite-difference^26^ and finite-element^27^ methods (FDM, FEM) and are the predominant method for designing acoustic devices in the GHz frequency range. Several commercial software tools^28^ allow the design of acoustic waveguides using such solvers, including domain reduction using Floquet (periodic) boundary conditions.

The most efficient approach to design a waveguide is to directly solve the source-free eigenmode problem on its 2D cross-section, the smallest domain that is necessary and sufficient to specify the problem (also referred to as ‘2 + 1D’). For lossless waveguides, the 2D formulation returns an orthogonal, complete set of modes (defined by an eigenfunction and eigenvalue pair corresponding to the field distribution and frequency) at a specified propagation constant (spatial wavelength). Formally, these are ‘resonator modes’ but represent waveguide modes. In contrast to the 2D case, solution of this ‘resonator’ eigen problem on a 3D volume returns all resonant modes, including those in higher order Brillouin zones, which act as unwanted artifacts of the 3D formulation of the 2D waveguide problem. A 2 + 1D FEM has been previously implemented to solve waveguide geometries in the ultrasound regime (referred to as the Semi-Analytic Finite Element, or SAFE, method)^29–32^.

Although FEM is more sophisticated and general than FDM (which it includes as a subset), FDM on a uniform grid has a number of strengths when it comes to the design of nano-scale photonic, and we believe by extension also phononic, devices and circuits. While optical FDM solvers are dominant in academic and commercial tools, an FDM solver has not been previously demonstrated for acoustic 2 + 1D simulations. First, a key design parameter in coupled-waveguide and coupled-cavity circuits is frequency or propagation constant splitting (supermode frequencies/propagation constants), which measures the strength of coupling of two structures. A uniform-grid FDM can provide these frequency/propagation constant differences between resonant/guided modes of coupled cavities/waveguides accurately with a coarse grid because absolute errors are common-moded out, whereas irregular meshes used in a FEM can introduce ‘grid induced’ or numerical fictitious detunings between nominally degenerate elements. These fictitious detunings result in physically incorrect coupling dynamics, and thus require a much finer grid (to achieve high absolute accuracy). Second, design of coupled structures often involves generating design curves by sweeping dimensions (e.g. spacing between coupled components). In these situations an unstructured FEM grid does not sweep consistently because the mesh adapts to the geometry, and may result in a non-smooth result vs. sweep parameter. An FDM implementation on a uniform grid can provide smooth, physically accurate results with proper grid alignment. In addition, FDM is simple and the implementation compact, allowing for straightforward addition of new functionalities such as other coordinate systems (e.g. cylindrical or elliptical) and addition of support for anisotropic materials. Last, the staggered grid FDM we implement naturally mates to electromagnetic solvers implemented on a Yee grid^33^ for optomechanical interaction simulations.

In this work, we develop and implement (and make the implementation freely available^34^) an acoustic waveguide mode solver which solves the linear isotropic elastic wave equation based on FDM (analogous to the SAFE method) which is comparably accurate to 3D FEM and more computationally efficient. We implement all common boundary conditions including free, fixed, and symmetry/anti-symmetry, as well as perfectly matched layers (PMLs) which permit efficient modeling of outward radiation and leaky modes. We validate the implementation’s accuracy from 1 MHz to 10 GHz to demonstrate its applicability in higher frequency regimes. A finite-difference method with staggered-grid discretization (identical to the staggered grid used in certain 3D acoustic solvers^35,36^) is chosen to preserve second-order accuracy in primary and derivative physical variables and enable direct alignment with the optical Yee grid^33^ for coupling to electromagnetics.

The solver finds the acoustic eigenmodes of a structure that is invariant along one Cartesian dimension. The source-free vectorial elastic wave equation in an inhomogeneous, isotropic, linear medium is formulated as an eigenvalue equation. This *β* to *ω* ‘resonator’ formulation is the ‘band structure’ form, convenient for computing the band diagram, with complex *ω* representing loss. The interchanged case with *β* as the eigenvalue is, however, a quadratic eigenvalue problem. This is notably different than the analogous electromagnetic problem which, due to Gauss’s Law, can be formulated as a linear eigenvalue problem with either *ω* or *β* given and the other variable as the eigenvalue. The acoustic *ω* to *β* ‘waveguide’ formulation can be linearized^37,38^, but this second type of implementation is beyond the scope of this work. A finite-difference discretization approach is then applied and transforms the continuous eigenvalue problem into a sparse matrix which can be solved by standard numerical sparse matrix eigen solver methods (here, the ‘eigs’ function in MATLAB^39^).

Notably, the use of 2 + 1D mode solvers in both optical and acoustic domains allows for accurate and efficient calculation of propagation parameters and coupling terms, which can be used as inputs to coupled mode theory or acousto-optic simulations^40,41^ for accuracy comparable to full 3D simulations but with orders of magnitude faster simulation times. Such fast simulation enables design approaches that are not practical with full 3D simulations, such as large parameter sweeps and optimizations. It also provides greater insight into the physics, and can be a building block enabling other physically insightful simulation techniques to be borrowed from optics, such as the film matching method^42,43^.

## Theory, Mathematical Framework, and Implementation of the Mode Solver

The mathematical basis for the eigenmode solver and its implementation are described in this section. We assume that the structure’s cross-section is invariant along one linear direction, $\hat{z}$, and that all materials are linear, isotropic, and time-invariant. The isotropic assumption is made for convenience and is not essential; the method can be applied to anisotropic media. This approach to solving for waveguide modes is analogous to electromagnetic mode solvers, but instead of an electric or magnetic field we solve for the elastic displacement field. Notably, in the elastic equations, there is no equivalent of Gauss’s Law and thus we solve for three field components and have three polarization families. In electromagnetics, Gauss’s Law reduces the eigen problem to two polarization mode families and specification of two field polarization components fully specifies the mode (e.g. *E*_*x*_, *E*_*y*_).

### Derivation of Isotropic Linear Elastic Wave Equation

We begin with Newton’s 2nd law written in a density formulation, the strain-displacement relation, and generalized Hooke’s law, respectively:

1 $$\rho {\partial }_{t}^{2}{\bf{u}}=\nabla \cdot \bar{\bar{\sigma }}$$

2 $$\bar{\bar{\varepsilon }}={\nabla }_{s}{\bf{u}}$$

3 $$\bar{\bar{\sigma }}={\mathbb{C}}:\bar{\bar{\varepsilon }}$$

where *ρ*(**r**, t) ≡ *ρ*(**r**) is the spatial distribution of material mass density, **u**(**r**, *t*) is the elastic displacement field, ∇⋅ is the tensor divergence, $\bar{\bar{\sigma }}({\bf{r}},t)$ is the stress tensor, $\bar{\bar{\varepsilon }}({\bf{r}},t)$ is the strain tensor, ∇_*s*_ is the symmetric spatial vector gradient, ${\mathbb{C}}({\bf{r}},t)\equiv {\mathbb{C}}({\bf{r}})$ is the fourth order stiffness tensor, and : denotes a tensor inner product^44^. Note that while stress and strain are second order tensors, due to the underlying symmetries they can be unwrapped as six-vectors following the Voigt notation ($\bar{\bar{\sigma }}={[{\sigma }_{xx}{\sigma }_{yy}{\sigma }_{zz}{\sigma }_{yz}{\sigma }_{xz}{\sigma }_{xy}]}^{T}$)^44^, which is the form used in this paper. The strain tensor has an analogous form. The operator ∇_*s*_ is the adjoint of the tensor divergence operator (${\nabla }_{s}^{\dagger }=-\nabla \cdot$). ${\mathbb{C}}$ is a symmetric operator (even in the presence of loss) due to our assumption of linear, time-invariant materials^44^, and we use the compact notation ∂_*i*_ to refer to the partial derivative ∂/∂*i*.

Substituting Eqs (2) and (3) into Eq. (1) in order to factor out the stress and strain tensors yields the linear elastic wave equation written in terms of the displacement field

4 $$\rho {\partial }_{t}^{2}{\bf{u}}=\nabla \cdot {\mathbb{C}}\,:{\nabla }_{s}{\bf{u}}\mathrm{.}$$

Invariance of *ρ* and ${\mathbb{C}}$ with time means that the system has a spectrum defined by a linear eigenvalue problem by setting ∂_*t*_ → *jω*. We define a weighted displacement field $\tilde{{\bf{u}}}\equiv \sqrt{\rho }{\bf{u}}$. This allows Eq. (4), a generalized eigenvalue problem with eigenvalue *ω* ^2^ when ∂_*t*_ → *jω*, to be recast as an ordinary eigenvalue problem with a Hermitian operator in the absence of loss. The elastic wave equation can be written as

5 $${\omega }^{2}\tilde{{\bf{u}}}=\frac{-1}{\sqrt{\rho }}\nabla \cdot {\mathbb{C}}\,:\,{\nabla }_{s}\frac{1}{\sqrt{\rho }}\tilde{{\bf{u}}}\mathrm{.}$$

This has the form of an eigenvalue equation (${\rm{\Lambda }}x=\bar{\bar{H}}x$), so we identify the eigenvalue as Λ ≡ *ω* ^2^, the eigenvector as $x\equiv \tilde{{\bf{u}}}$, and the symmetric elastic resonance operator $\bar{\bar{H}}\equiv -{\rho }^{-\mathrm{1/2}}\nabla \cdot {\mathbb{C}}\,:\,{\nabla }_{s}{\rho }^{-\mathrm{1/2}}$. To clarify this expression, the differential operators (in the form that operate on the reduced, six-vector notation used for $\bar{\bar{\sigma }}$ and $\bar{\bar{\varepsilon }}$) can be written in matrix form as

6 $${\rm{\nabla }}\cdot =-{{\rm{\nabla }}}_{s}^{\dagger }=[\begin{array}{cccccc}{{\rm{\partial }}}_{x} & 0 & 0 & 0 & {{\rm{\partial }}}_{z} & {{\rm{\partial }}}_{y}\\ 0 & {{\rm{\partial }}}_{y} & 0 & {{\rm{\partial }}}_{z} & 0 & {{\rm{\partial }}}_{x}\\ 0 & 0 & {{\rm{\partial }}}_{z} & {{\rm{\partial }}}_{y} & {{\rm{\partial }}}_{x} & 0\end{array}].$$

To this point, we have defined the acoustic resonator problem (in 3D). Next, when solving for eigenmodes of a structure with *z*-invariant geometry, by Fourier transformation along *z* the *z*-directed derivative yields the propagation constant (∂_*z*_ = − *jβ*). This reduces the problem to one on the cross-sectional plane and ensures that only modes with the specified propagation constant *β* will be returned by the solver.

Next, making the isotropic assumption, the stiffness tensor (in the form that operates on the six-vector notation) can be reduced to

7 $${\mathbb{C}}=[\begin{array}{cccccc}{c}_{11} & {c}_{12} & {c}_{12} & 0 & 0 & 0\\ {c}_{12} & {c}_{11} & {c}_{12} & 0 & 0 & 0\\ {c}_{12} & {c}_{12} & {c}_{11} & 0 & 0 & 0\\ 0 & 0 & 0 & {c}_{44} & 0 & 0\\ 0 & 0 & 0 & 0 & {c}_{44} & 0\\ 0 & 0 & 0 & 0 & 0 & {c}_{44}\end{array}]$$

where *c*_*ij*_(**r**, *t*) ≡ *c*_*ij*_(**r**) are the Voigt notation stiffness coefficients which can be defined in terms of the Lamé parameters^44^ as *c*_11 _= *λ *+ 2*μ*, *c*_12 _= *λ*, *c*_44 _= *μ*.

### Discretization Scheme

In order to solve the eigenvalue problem in an arbitrary cross-section geometry (*ρ*, ${\mathbb{C}}$) numerically, Eq. (5) is discretized over a finite 2D domain terminated by boundary conditions and is cast as a matrix eigenvalue problem. An appropriate 3D grid is first formulated which accurately captures the physics to second-order accuracy in the discretization and the grid is then collapsed to the 2D simulation domain. The collapse is performed such that the cross-sectional locations of all grid points are unchanged but are all referenced to a single *z* position. The *z*-derivatives, which would be performed between two points in the 3D case, amount to multiplying a single grid point by −*jβ* in the 2D case. Formally, for a *z*-invariant structure on a 3D grid, ${\partial }_{z}\to -\,j\beta \,\sin (\beta {\rm{\Delta }}z/2)/(\beta {\rm{\Delta }}z/2)\exp (-\,j\beta {\rm{\Delta }}z/2)$ ^45^, but as Δ*z *→ 0 then ∂_*z *_→ −*jβ*.

**Figure 1:**
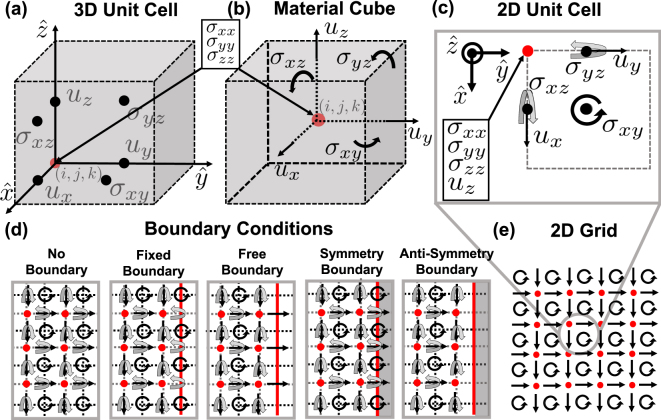
(**a**) Unit cell of the 3D discretization grid used in this paper, where the unit cell coordinates (*i*, *j*, *k*) define the corner of the unit cell and are co-located with the principal stresses. Each point is denoted with the quantities which are sampled at that point. (**b**) Discrete ‘material cube’ which provides the basis for an intuitive choice of discretization grid. The ‘material cube’ is offset from the unit cell by a half-step along each dimension. (**c**) 2D unit cell which can be obtained by collapsing the 3D unit cell along the *z*-dimension. (**d**) Representative implementation of the boundary conditions, including choice of location (red line) and values sampled on the boundary (or removed). (**e**) 2D discretization grid with schematic depiction of component grid locations.

The discretization grid used is depicted in Fig. 1. This grid is based on physical intuition from solid mechanics: the state of an element cube [Fig. 1(b)] is primarily described by the principal stresses (*σ*_*xx*_, *σ*_*yy*_, *σ*_*zz*_), which we define to reside at the center of the cube. The principal stresses lead to the deformation of the faces of the cube, such that the center of each face is the sampling location of the associated surface-normal displacement. If grid point (*i*, *j*, *k*) is associated with the principal stresses (and the center of the cubic volume elements), the corresponding displacements are *u*_*x*_ : (*i* + 1/2, *j*, *k*), *u*_*y*_ : (*i*, *j* + 1/2, *k*), and *u*_*z*_: (*i*, *j*, *k* + 1/2). An appropriate grid for the shear stresses is found to be a further half-step offset, such that shear stresses are located at *σ*_*xy*_ : (*i* + 1/2, *j* + 1/2, *k*), *σ*_*xz*_ : (*i* + 1/2, *j*, *k* + 1/2), and *σ*_*yz*_ : (*i*, *j* + 1/2, *k* + 1/2). The strain grid is co-located with the stress grid (*ε*_*mn*_ is co-located with *σ*_*mn*_) and the material grid (${\mathbb{C}}$, *ρ*) is located at the cube centers (*i*, *j*, *k*). The finite-difference operators that approximate the spatial partial derivatives transform quantities from the stress/strain grid to the displacement grid and back, as expected and desired. Because isotropic materials are assumed, the material operator ${\mathbb{C}}$ does not induce a change of grid coordinates (the stress at a specific grid point is only related to the strains at the same grid point). This method can also be extended to anisotropic materials at the cost of additional complexity in the ${\mathbb{C}}$ matrix.

The staggered-grid scheme used here is analogous to the Yee grid^33^, commonly used in electromagnetic solvers, which preserves second-order accuracy for all fields due to centered differencing (when all materials/coefficients vary spatially on the scale of the discretization). Our discretization grid is slightly more complicated in that the elastic displacement field and shear stress directly replicate a Yee grid, while the principal stresses occupy an additional position at the center of each ‘cube’.

### Finite Difference Operators

Centered differences in 2^nd^ order differential equations can be formed by appropriate combinations of forward and backward differences on a staggered grid^45^. Denoting forward differences as ${\tilde{\partial }}_{i}$ and backward differences as ${\hat{\partial }}_{i}$, the differential operators can be rewritten in terms of forward and backward differences (where the propagation constant has been substituted for ∂_*z*_) as

8 $${{\rm{\nabla }}}_{s}=[\begin{array}{ccc}{\hat{{\rm{\partial }}}}_{x} & 0 & 0\\ 0 & {\hat{{\rm{\partial }}}}_{y} & 0\\ 0 & 0 & -j\beta \\ 0 & -j\beta  & {\mathop{{\rm{\partial }}}\limits^{ \sim }}_{y}\\ -j\beta  & 0 & {\mathop{{\rm{\partial }}}\limits^{ \sim }}_{x}\\ {\mathop{{\rm{\partial }}}\limits^{ \sim }}_{y} & {\mathop{{\rm{\partial }}}\limits^{ \sim }}_{x} & 0\end{array}]$$

9 $${\rm{\nabla }}\cdot =[\begin{array}{cccccc}{\mathop{{\rm{\partial }}}\limits^{ \sim }}_{x} & 0 & 0 & 0 & -j\beta  & {\hat{{\rm{\partial }}}}_{y}\\ 0 & {\mathop{{\rm{\partial }}}\limits^{ \sim }}_{y} & 0 & -j\beta  & 0 & {\hat{{\rm{\partial }}}}_{x}\\ 0 & 0 & -j\beta  & {\hat{{\rm{\partial }}}}_{y} & {\hat{{\rm{\partial }}}}_{x} & 0\end{array}].$$

It should be noted that, because ${{\tilde{\partial }}_{i}}^{\dagger }=-{\hat{\partial }}_{i}$, ∇_*s*_ and −∇⋅ remain exact adjoints in discrete form (${\nabla }_{s}^{\dagger }=-\nabla \cdot$).

### Matrix Operator Construction

The resonance operator can be rewritten as a matrix, found by discretizing the displacement field and unwrapping the field into a single column vector. A convenient ordering is $\tilde{{\bf{u}}}={[\{{u}_{x}\}\{{u}_{y}\}\{{u}_{z}\}]}^{T}$ where {*u*_*i*_} is a vectorized form that is unwrapped such that adjacent points in the *x*-dimension are adjacent in the vector. Thus, each vector component is unwrapped in the *x *− *y* plane along the *x*-dimension first and the components are then concatenated. This results in a vector of approximate length 3*n*_*x*_ *n*_*y*_ from an *n*_*x *_× *n*_*y *_× 3 matrix. A similar method is used to unwrap the stress tensor in a vector of approximate length 6*n*_*x*_ *n*_*y*_ of the form $\bar{\bar{\sigma }}={[\{{\sigma }_{xx}\}\{{\sigma }_{yy}\}\{{\sigma }_{zz}\}\{{\sigma }_{yz}\}\{{\sigma }_{xz}\}\{{\sigma }_{xy}\}]}^{T}$ with the strain tensor having an equivalent form. Note that the different components *σ*_*ij*_ and *u*_*i*_ are of different lengths since they are sampled at different locations.

The differential operator hereby becomes a *matrix* operator where each entry in Eqs (7), (8), (9) is now a block matrix which relates one component to another. After populating the ∇⋅, ${\mathbb{C}}$, and ∇_*s*_ matrices with the block matrices, they are multiplied together to give the matrix operator $\bar{\bar{H}}$. We here note that the matrix, currently in a complex-valued Hermitian form in the absence of loss (due to *jβ* terms), can be cast as a real symmetric matrix $\bar{\bar{H}}^{\prime}$ (a special case of Hermitian matrices) by defining a modified eigenvector form $\tilde{{\bf{u}}}^{\prime} \equiv [{\tilde{u}}_{x},\,{\tilde{u}}_{y},\,-j{\tilde{u}}_{z}]$, i.e. the *u*_*z*_ component will be in quadrature with the *u*_*x*_, *u*_*y*_ components. Hermitian matrices have real eigenvalues (energy conservation) and their eigenvectors form a complete, orthogonal set. Symmetric matrices, as an additional trait, have eigenvectors that can be chosen to be entirely real. If loss is present, then this modified matrix form will be complex symmetric rather than real symmetric and we would expect to obtain complex eigenvalues and eigenmodes with an unconjugated orthogonality condition that is not defined by energy conservation. Defining the modified operator matrix $\bar{\bar{H}}^{\prime}$ such that ${\omega }^{2}\tilde{{\bf{u}}}^{\prime} =\bar{\bar{H}}^{\prime} \tilde{{\bf{u}}}^{\prime}$, we can calculate this modified operator matrix by recognizing that $\tilde{{\bf{u}}}^{\prime} =\bar{\bar{R}}\tilde{{\bf{u}}}$ and $\bar{\bar{H}}^{\prime} =\bar{\bar{R}}\bar{\bar{H}}{\bar{\bar{R}}}^{-1}$ where $\bar{\bar{R}}$ is a diagonal matrix with ${\rm{diag}}(\bar{\bar{R}})=\mathrm{[1,}\,\mathrm{1,}\,-j]$. We can then write the modified operator matrix as

10 $$\bar{\bar{H}}^{\prime} =\frac{-1}{\sqrt{\rho }}[\begin{array}{ccc}{\mathop{{\rm{\partial }}}\limits^{ \sim }}_{x}{c}_{11}{\hat{{\rm{\partial }}}}_{x}-{c}_{44}{\beta }^{2}+{\hat{{\rm{\partial }}}}_{y}{c}_{44}{\mathop{{\rm{\partial }}}\limits^{ \sim }}_{y} & {\mathop{{\rm{\partial }}}\limits^{ \sim }}_{x}{c}_{12}{\hat{{\rm{\partial }}}}_{y}+{\hat{{\rm{\partial }}}}_{y}{c}_{44}{\mathop{{\rm{\partial }}}\limits^{ \sim }}_{x} & -\beta {\mathop{{\rm{\partial }}}\limits^{ \sim }}_{x}{c}_{12}-\beta {c}_{44}{\mathop{{\rm{\partial }}}\limits^{ \sim }}_{x}\\ {\mathop{{\rm{\partial }}}\limits^{ \sim }}_{y}{c}_{12}{\hat{{\rm{\partial }}}}_{x}+{\hat{{\rm{\partial }}}}_{x}{c}_{44}{\mathop{{\rm{\partial }}}\limits^{ \sim }}_{y} & {\mathop{{\rm{\partial }}}\limits^{ \sim }}_{y}{c}_{11}{\hat{{\rm{\partial }}}}_{y}-{c}_{44}{\beta }^{2}+{\hat{{\rm{\partial }}}}_{x}{c}_{44}{\mathop{{\rm{\partial }}}\limits^{ \sim }}_{x} & -\beta {\mathop{{\rm{\partial }}}\limits^{ \sim }}_{y}{c}_{12}-\beta {c}_{44}{\mathop{{\rm{\partial }}}\limits^{ \sim }}_{y}\\ \beta {c}_{12}{\hat{{\rm{\partial }}}}_{x}+\beta {\hat{{\rm{\partial }}}}_{x}{c}_{44} & \beta {c}_{12}{\hat{{\rm{\partial }}}}_{y}+\beta {\hat{{\rm{\partial }}}}_{y}{c}_{44} & -{\beta }^{2}{c}_{11}+{\hat{{\rm{\partial }}}}_{y}{c}_{44}{\mathop{{\rm{\partial }}}\limits^{ \sim }}_{y}+{\hat{{\rm{\partial }}}}_{x}{c}_{44}{\mathop{{\rm{\partial }}}\limits^{ \sim }}_{x}\end{array}]\frac{1}{\sqrt{\rho }}.$$

We remind the reader here that, even for complex finite difference operators and complex discretization Δ*x*, ${\hat{{\rm{\partial }}}}_{i}^{T}=-{\mathop{{\rm{\partial }}}\limits^{ \sim }}_{i}$ so that this modified matrix $\bar{\bar{H}}^{\prime}$ is symmetric. It is additionally real if both material parameters (*c*_*ij*_) and finite difference operators (${\hat{\partial }}_{i},{\tilde{\partial }}_{i}$) are real, corresponding to no material loss and no complex coordinate stretching (radiation absorbing boundaries), respectively.

### Modal Orthogonality

The orthogonality condition for vectors (in the sense of 1D tensors) is ${{\bf{x}}}_{i}^{\dagger }{{\bf{x}}}_{j}\equiv {|{{\bf{x}}}_{i}|}^{2}{\delta }_{ij}$ where the vector norm is defined as $|{{\bf{x}}}_{i}|\equiv {({{\bf{x}}}_{i}^{\dagger }{{\bf{x}}}_{i})}^{1/2}$. These two equations correspond to ∫∫**x**_*i*_(**r**_*T*_)^*^ ⋅ **x**_*j*_(**r**_*T*_)*dA* = |**x**_*i*_||**x**_*j*_|*δ*_*ij*_ and $|{{\bf{x}}}_{i}|={(\iint {{\bf{x}}}_{i}^{\ast }({{\bf{r}}}_{T})\cdot {{\bf{x}}}_{i}({{\bf{r}}}_{T})dA)}^{1/2}$ when **x** represents a 3-vector field in a 2D domain. Noting that that ${\tilde{{\bf{u}}}}_{i}^{\text{'}\ast }\cdot {\tilde{{\bf{u}}}}_{j}^{\text{'}}=\rho {{\bf{u}}}_{i}^{\ast }\cdot {{\bf{u}}}_{j}$, we can then replace ${\bf{x}}\to \tilde{{\bf{u}}}^{\prime}$ and write the orthogonality condition and normalization as

11 $$\iint \rho {{\bf{u}}}_{i}^{\ast }\cdot {{\bf{u}}}_{j}dA=|{\tilde{{\bf{u}}}}_{i}||{\tilde{{\bf{u}}}}_{j}|{\delta }_{ij}$$

12 $$|{\tilde{{\bf{u}}}}_{i}|={(\iint \rho {{\bf{u}}}_{i}^{\ast }\cdot {{\bf{u}}}_{i}dA)}^{\frac{1}{2}}\mathrm{.}$$

Noting that the particle velocity field is ***v***_*i*_ ≡ ∂_*t*_ **u**_*i*_ = *jω*_*i*_ **u**_*i*_, we can substitute **u**_*i*_ = −*j* **v**_*i*_/*ω*_*i*_ into Eqs (11) and (12) to obtain measures of the kinetic energy $({E}_{K}=1/2\iint \rho {|{\bf{v}}|}^{2}dA)$. Alternatively, because the potential energy $(V=1/2\iint {\bar{\bar{\varepsilon }}}^{\dagger }{\mathbb{C}}\bar{\bar{\varepsilon }}dA)$ is equivalent to the kinetic energy when averaged^44^, this gives the orthogonality condition that would have been obtained if the wave equation were formulated with $\bar{\bar{\varepsilon }}$ as the free variable. This is analogous to the electromagnetic case, where resonator problems use an energy formulation to determine modal orthogonality^46^.

### Boundary Conditions

Four boundary conditions are implemented: fixed boundary (**u**|_*Bound*_ = 0), free boundary ($\bar{\bar{\sigma }}\cdot \hat{{\bf{n}}}=0$), symmetry boundary (∂_*n*_ *u*_*n*_ = 0, **u**_*t*_|_*Bound*_ = 0), and anti-symmetry boundary (*u*_*n*_|_*Bound *_= 0, ∂_*n*_ **u**_*t *_= 0). Perfectly matched layers (PMLs) are radiation-absorbing regions which permit simulation of radiating structures^47^ and are also implemented. For all boundary conditions, we locate the boundary such that the boundary-normal displacements (*u*_*n*_), corresponding out-of-plane shear stresses (*σ*_*nz*_), and in-plane shear stresses (*σ*_*xy*_) lie on the boundary. The choice of boundary location is depicted in Fig. 1(d) as the location of the red line. We additionally allow for vacuum (no material) in the simulation domain.

The fixed boundary condition requires the displacement field be set to zero on the boundary. An efficient and computationally stable implementation is to remove the corresponding boundary-normal displacements *u*_*n*_ (since they are the only ones coincident with the boundary) from the matrix operator and solution vector, which is the method used here. We validate this boundary condition in Sec. 2.3 by confirming that a beam with fixed boundary conditions returns the correct eigenmode shapes and frequencies.

For the free surface boundary condition, the boundary-normal stress components must go to zero at the boundary. In a similar manner to the implementation of the fixed boundary condition, we remove the shear stresses *σ*_*nz*_, *σ*_*xy*_ on the boundary. This boundary condition is also validated in Sec. 2.3 by confirming that the eigenmode shapes and frequencies are accurately calculated.

Symmetry and anti-symmetry boundary conditions are hybrids of free and fixed boundary conditions. Similar to the case in electromagnetics, symmetry of the normal displacement corresponds to anti-symmetry of the transverse displacement, while anti-symmetry of the normal displacement corresponds to symmetry of the transverse displacement. A symmetry (anti-symmetry) boundary therefore refers to symmetry (anti-symmetry) of the normal displacement and anti-symmetry (symmetry) of the transverse displacement. The proper implementation of these boundary conditions is validated in Sec. 2.3 by comparison of the free beam modes calculated with and without the symmetry/anti-symmetry boundary conditions.

We enable vacuum within the simulation domain by removing both stress and displacement grid points within the vacuum region. We also remove the stress sampled on the vacuum-material boundary as appropriate. This exactly emulates a free surface boundary condition along the interface, as desired.

We additionally implement PMLs in order to support simulation of leaky modes. The PML is not formally a boundary condition and is instead a region within the computational domain^47–50^. A desired use for this acoustic mode solver is to estimate propagation losses of a particular acoustic mode in a waveguide, and a key loss mechanism can be radiation loss (analogous to a distributed version of clamping loss in resonators, e.g. cantilevers). We refer to acoustic modes exhibiting radiation loss as a leaky modes, which can still be considered confined modes when the radiation loss rate is much smaller than the oscillation frequency.

The PML boundary condition can be implemented as a complex coordinate stretching near the boundary^50^. This can be achieved by making the grid discretization components Δ*x* and Δ*y* complex within the PML region. A linear coordinate stretching was used of the form

13 $${\rm{\Delta }}{x}_{PML}={\rm{\Delta }}x(1-j\alpha |\frac{x-{x}_{PML,begin}}{{x}_{PML,end}-{x}_{PML,begin}}|){x}_{PML,end}\ge x\ge {x}_{PML,begin}$$

where *α* is the scaling parameter of the PML. To demonstrate the accuracy of the PMLs implemented in this solver, we constructed a leaky acoustic waveguide that tunnels radiation across a barrier into the continuum on both sides in Sec. 2.5.

## Mode Solver Validation and Demonstration

### Mathematical Validation: Matrix Properties

**Figure 2:**
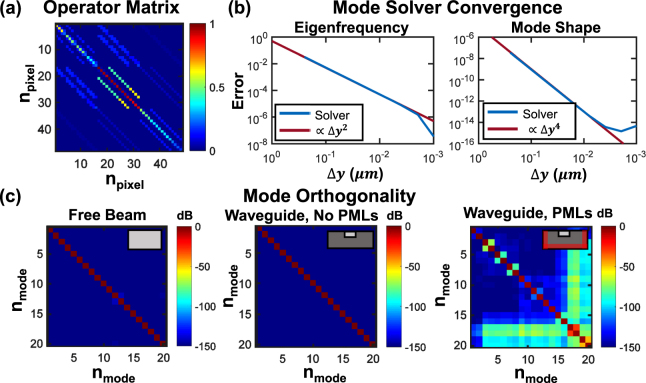
Validation of resonance operator matrix properties. (**a**) Example of the operator matrix. (**b**) Convergence tests of the mode solver: the 20 dB/decade slope for eigenfrequency error (40 dB/decade for mode shape) indicates second-order accuracy. (**c**) Tests of modal orthogonality in three configurations, supporting the Hermitian nature of the resonance operator in the absence of PMLs and the modified orthogonality relation for the lossy case.

To test our discretization scheme, we examine the properties of resonance operator matrices generated by the mode solver code. We implement a trial cross-section with two free and two fixed boundary conditions on a 4 × 4 pixel grid to allow the matrix structure to be visualized in Fig. 2(a). We additionally verify in Fig. 2(b) second order scaling of the error with discretization. For this case, we chose an anti-symmetric Lamb wave with a 10 μm wavelength in a 1 μm thick free slab and varied the discretization along the slab thickness direction. The calculated eigenfrequency and mode shape errors are relative to the analytical solutions, described further in Sec. 2.2. Our convergence plots indicate that the mode solver eigenfrequency error scales as *ε* ∝ (Δ*y*)^2^, i.e. that our mode solver is second-order accurate as expected. The mode shape error scales as *ε* ∝ (Δ*y*)^4^ due to the squaring in the mode shape error formula. We additionally verified that, in the absence of material loss or PMLs, the resonance operator is real and symmetric after discretization using a 200 × 200 pixel simulation. We also confirmed that the operator is complex symmetric when PMLs are used. Other hallmarks of Hermitian operators are real eigenvalues, orthogonal eigenvectors, and that the eigenvectors form a complete basis. We have shown an example demonstrating the orthogonality of the eigenvectors in both a free beam and a waveguide without PMLs, as well as the modified orthogonality when PMLs are implemented $(\iint {\tilde{{\bf{u}}}}_{i}^{^{\prime} }\cdot {\tilde{{\bf{u}}}}_{j}^{^{\prime} }dA)$, in Fig. 2(c).

### Analytical Validation: Free Slab Waveguide

**Figure 3:**
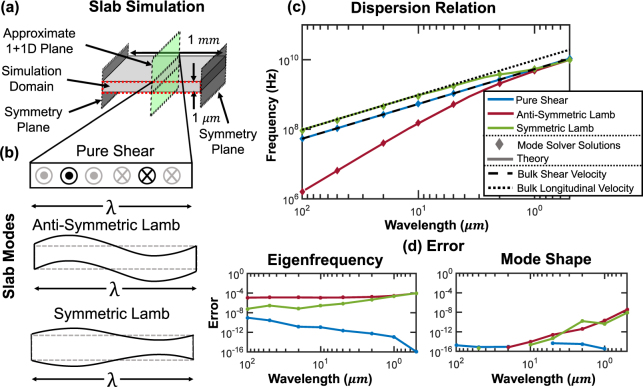
Three acoustic modes (pure shear, anti-symmetric Lamb, and symmetric Lamb) in a 1 μm thick silicon slab were simulated and compared to the theoretical modal distributions and frequencies: (**a**) Configuration schematic. (**b**) Illustration of the three acoustic modes simulated. (**c**) Modal dispersion curves; solid lines are theoretical dispersions and diamond markers are mode solver solutions (shear and longitudinal bulk wave asymptotes also shown). (**d**) Relative error in frequency and mode shape between the mode solver and theory.

We choose a slab waveguide, which can be analytically solved, to compare the proposed mode solver with elastic wave theory. The simulation implementation and results are shown in Fig. 3. We investigate three fundamental acoustic waves in a thin plate [Fig. 3(b)], namely pure shear waves, anti-symmetric Lamb waves, and symmetric Lamb waves^44^. We calculate the theoretical dispersion relations [Fig. 3(c)] of these waves compared with the mode solver solutions as the wavelength is varied from 100 μm to 0.5 μm to span the thin plate regime and into the thick plate (infinite half-space) regime. For the mode solver solutions, we use a plate which is much wider than its thickness (1 × 1000 μm) and sample the acoustic mode distribution at the center of the plate to approximate the theoretical 1 + 1D case. We then calculate the error of the mode solver relative to theory, both in terms of mode shape and eigenfrequency, in Fig. 3(d). The mode shape error used here is formulated as ${\varepsilon }_{MS}=1-({|\iint {U}_{MS}\cdot {U}_{o}^{\ast }{\rm{dA}}|}^{2})/(\iint {|{U}_{MS}|}^{2}{\rm{dA}}\iint {|{U}_{o}|}^{2}{\rm{dA}})$ where *U*_*o*_ is the true mode shape and *U*_*MS*_ is the mode shape found by the solver. For the slab case, we integrate over only a single dimension as the mode shapes are invariant along the second cross-sectional dimension. For the general case, the integration is carried out over the entire cross-section. The mode solver reproduces the key aspects of these acoustic waves with <0.01% error across a range of wavelengths which captures the thick, thin, and wavelength-scale plate thickness regimes, indicating that the mode solver faithfully models linear elastic physics.

### Numerical Validation: Free and Fixed Beam Waveguides

**Figure 4:**
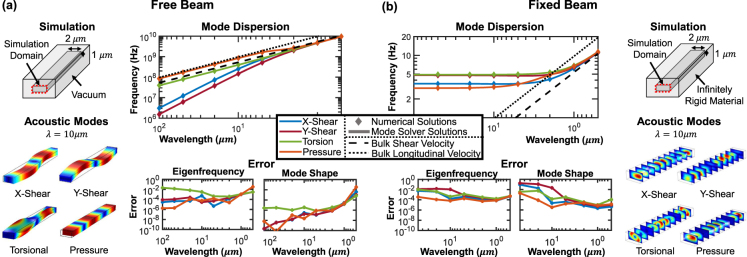
Four acoustic modes (*x*- and *y*-shear, torsion, and pressure waves) in suspended (**a**) and fixed (**b**) silicon beams were simulated across a wavelength range of 100 μm to 0.5 μm in both the mode solver and the 3D solver. Shown in each box for the suspended and fixed configurations are a schematic of the simulation implementation, depictions from the 3D solver of the acoustic modes, modal dispersions, and relative error between the mode solver and the numerical solver. The solid lines in the dispersion plots are the mode solver solutions and the diamond markers are the numerical solver solutions.

We now consider a rectangular beam waveguide (in both suspended and fixed, i.e. encased in an infinitely rigid medium, configurations) to compare the proposed mode solver with numerical solutions obtained from a commercial 3D solver^28^ (which returns a considerably larger solution space and is correspondingly more computationally intensive). We investigate the lowest order mode in each of four basic mode families: vertically (*y*) polarized shear waves, horizontally (*x*) polarized shear waves, torsional waves, and pressure waves. We choose as our cross-section a 2 × 1 μm silicon beam so as to avoid a degeneracy of the *x*- and *y*-shear modes. The same geometry is implemented in the commercial 3D solver with the beam length chosen equal to the simulated wavelength and Floquet periodic boundary conditions applied to the *z*-oriented boundaries. Both solvers are then used to find these four acoustic modes and corresponding frequencies across a wavelength range of 100 μm to 0.5 μm. These comparisons are made in Fig. 4 where we depict the comparative error of the mode solver relative to the 3D solver in terms of frequency and mode shape. For the majority of wavelengths, both the mode shape and frequency are very close (<1%) to the 3D solver results, defined as the reference value. The increased error at large wavelengths in the fixed beam, and lack of data at 100 μm, is due to difficulty in simulating these modes in the commercial solver.

### Symmetry/Anti-symmetry Boundary Condition Validation

**Figure 5:**
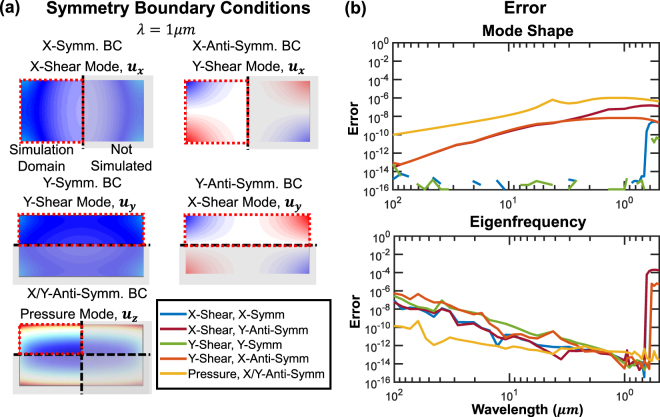
Three acoustic modes (*x*- and *y*-shear and pressure waves) are simulated in a suspended silicon beam in a truncated simulation domain using symmetric and anti-symmetric boundary conditions and compared with the same modes simulated in the full simulation domain. (**a**) Schematic depictions of the symmetric and anti-symmetric boundary conditions and simulated mode shapes. (**b**) Comparison of mode shape and eigenfrequency error between the modes obtained with the full simulation domain, and the truncated simulation domain using symmetry/anti-symmetry boundary conditions. Increased errors at the shortest wavelengths are due to the mode solver returning mixed modes when the eigenfrequencies are nearly degenerate.

Having demonstrated the accuracy of the solver, we can use the free beam modes as a basis for comparison with modes computed in a reduced simulation domain that takes advantage of geometrical symmetries. For this case we halve (or quarter) the simulation domain and use a symmetry/anti-symmetry boundary condition on the cut plane to recreate the same mode. We have chosen to use both boundary conditions to recreate the same mode as a redundant test of the boundary conditions, and the pressure mode is chosen as a demonstration that two orthogonal boundary conditions can be used at the same time without loss of accuracy. This comparison is shown in Fig. 5, where examples of the modes and symmetry conditions are shown in Fig. 5(a) and the errors are shown in Fig. 5(b). The jump in error in both eigenfrequency and mode shape at small wavelengths is due to the simulated modes being nearly degenerate in frequency at these wavelengths, causing the solver to return mixed mode shapes and eigenfrequencies when the full simulation domain is used. The halved/quartered simulation domains did not have mixing due to the imposed symmetry requirements.

### Solver Demonstration: Evanescent Waveguiding, Leaky Modes, and PMLs

We now construct an acoustic waveguide^7,51,52^ formed of two materials (a ‘core’ and a ‘cladding’) and find evanescently confined acoustic modes. Adding a radiation layer (formed of core material) to the waveguide cross-section creates a leaky acoustic waveguide that tunnels radiation across a (cladding) barrier into the radiation-mode continuum on both sides (see analogous optical waveguide pg. 93^53^). Implementation of PMLs on the simulation domain edges then allows outward radiating waves to be absorbed without reflection, enabling the simulation of leaky modes analogous to electromagnetic solvers^54^ and calculation of waveguide mode radiation losses.

**Figure 6:**
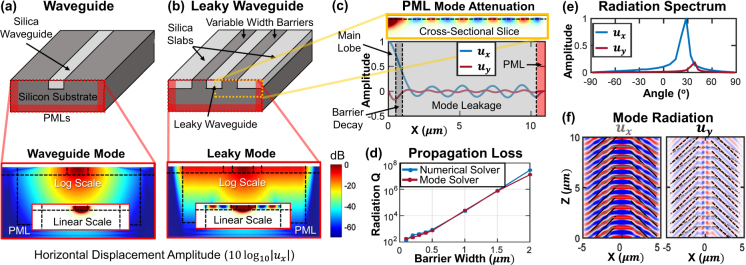
An embedded strip acoustic waveguide (1 × 0.4 μm) is simulated to demonstrate evanescent confinement, wave-guiding, radiation tunneling, leaky modes, and PML operation. (**a**) Schematic depiction of an acoustic waveguide and log-scale, cross-sectional plot of the horizontal displacement of the quasi-Love wave guided mode with linear scale inset. (**b**) The same waveguide after silica slabs have been added (with a 0.5 μm silicon barrier) with plots of the leaky mode. (**c**) An extended 1D slice of the leaky acoustic mode in (**b**) showing the displacement amplitude. (**d**) Propagation loss comparison between the 3D solver and the mode solver. (**e**) Fourier transform of displacement amplitudes in the leaky section of the 1D slice from (**c**), demonstrating coupling into two radiation modes. (**f**) Mode propagation and radiated wavefronts (shown schematically) for both horizontal and vertical displacement components.

The configuration, modal amplitude plots, radiation patterns, and calculated radiation losses are shown in Fig. 6. First, an evanescently guiding waveguide is constructed [Fig. 6(a)] by embedding a strip of silica within a silicon substrate. This waveguide supports two ‘polarizations’ of acoustic modes, analogous to the Love and Rayleigh surface waves^44^. The former wave is chosen and the depicted log-scale plot of the horizontal displacement amplitude demonstrates that the wave is evanescently confined. We then add silica slabs to either side of the silica waveguide, separated by variable width silicon barriers, which cause the waveguide and guided acoustic modes to couple to the slab radiation continuum on both sides and become ‘leaky’ [Fig. 6(b)]. Oscillation of the field within the silica layer can be seen in the inset, and non-decaying intensity within the leaky region can be seen in the log-scale plot; both are indicative of the radiative loss of the guided mode into the adjacent silica layers.

An extended simulation domain with a longer silica layer further demonstrates the presence of this mode leakage in Fig. 6(c) where a cross-sectional slice plots both horizontal and vertical displacement amplitudes within the different regions. The mode displacement is predominantly confined in the waveguide with an evanescent tail in the silicon barrier. Some of the acoustic energy tunnels across the barrier into the silica slab, where it becomes oscillatory, and then enters the PML and is attenuated as expected. A comparative plot of this propagation loss, as a function of silicon barrier width, is shown in Fig. 6(d) as numerical validation of the PML accuracy. By taking a Fourier transform of the mode leakage section we show that two primary radiation field components are excited in the leaky wave [Fig. 6(e)], corresponding to quasi-Love (*u*_*x*_) and quasi-Rayleigh (*u*_*y*_) waves in the silica slab. Figure 6(f) shows a cross-section in the *x* − *z* plane depicting the *u*_*x*_ (left) and *u*_*y*_ (right) displacement amplitudes, where the wavefronts have been outlined for both polarizations and overlaid on both plots (gray for horizontal displacement, black for vertical). The figure shows a waveguide mode that has two leakage channels – quasi-Love and quasi-Rayleigh – with different radiation angles. Multiple discrete radiative channels are not present in electromagnetic leaky modes. We suggest naming this type of mode ‘bi-leaky’. It may find utility in design of phononic circuits (e.g. filters^55^).

## Conclusion

We have developed an eigenmode solver based on a finite-difference scheme on a staggered-grid for the linear, isotropic elastic wave equation. We have verified the accuracy of the solver by comparison with both theory and a 3D numerical solver (COMSOL). We have also used these comparisons to demonstrate correct implementation of fixed and free boundary conditions. We then used a leaky acoustic waveguide to demonstrate the solver’s ability to simulate evanescent guiding, leaky modes, and numerically accurate PMLs. We expect that the mode solver will be of utility in the design of on-chip acoustic wave devices. It is particularly suited to interfacing with electromagnetic solvers on the Yee grid for applications based on acousto-optics and optomechanics. Potential future extensions to this solver include support for material anisotropy, a cylindrical coordinate system formulation for simulating ring and disk resonators, co-integration with an optical mode solver for optomechanics applications, and reformulation of the eigenvalue problem to solve for *β* as the eigenvalue with *ω* provided, similar to electromagnetic mode solvers for waveguides.

### Data Availability

The datasets generated and analyzed during the current study are available from the corresponding author on reasonable request.
